# Supplementary material for: Altered microRNA Transcriptome in Cultured Human Liver Cells upon Infection with Ebola Virus
Source: Int J Mol Sci. 2021 Apr 6;22(7):3792. doi: 10.3390/ijms22073792 (PMC8038836; doi:10.3390/ijms22073792)
Supplement: Supplementary file 1 [file ijms-22-03792-s001.zip › Supplementary_File/D_ KEGG_Analysis_Results/16-30nt_pathway_Makona-96h-Huh7_vs_Control-96h-Huh7_up.mature_mirna_targets/hsa_GeneRatioDotPlot.pdf]

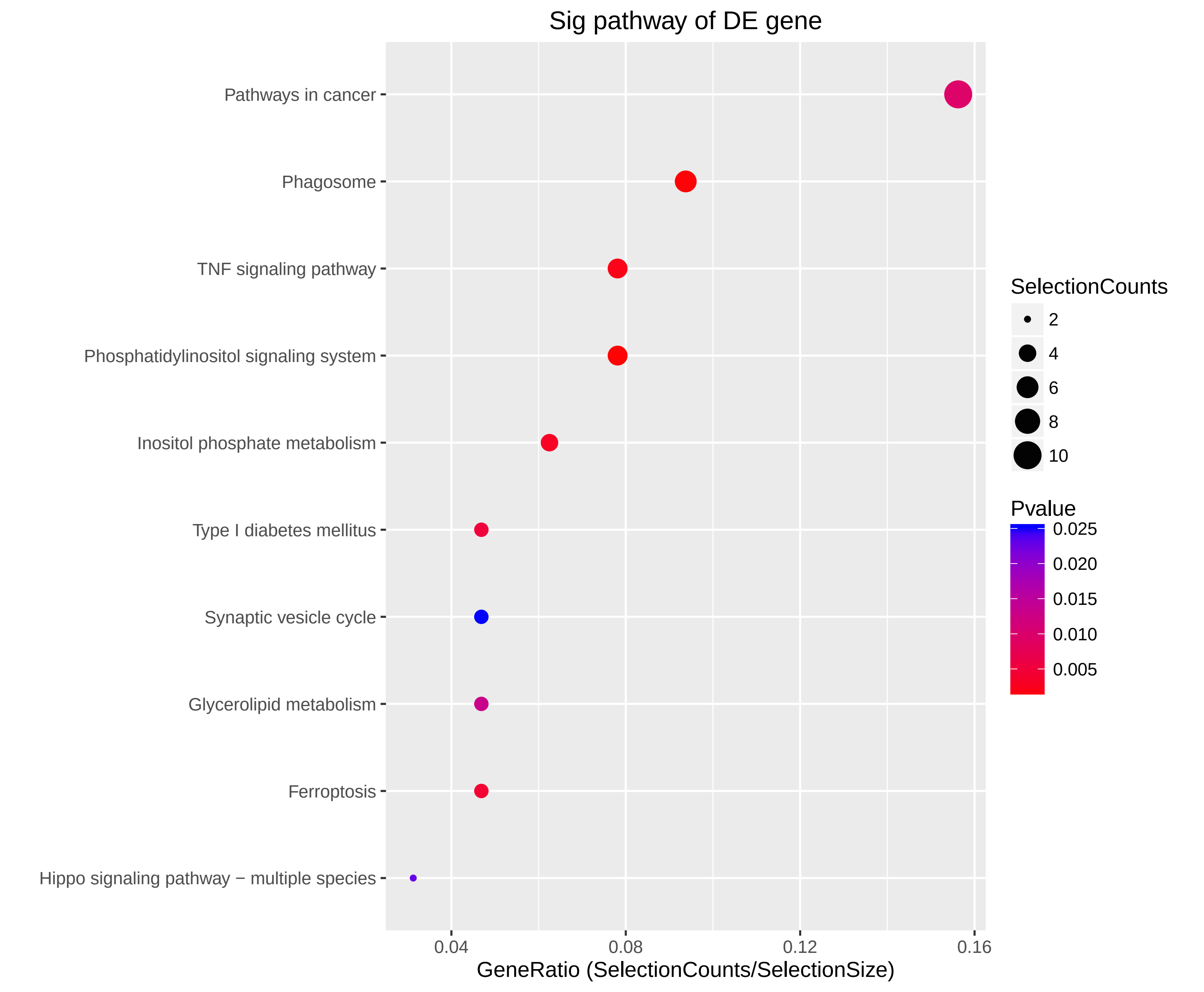
Sig pathway of DE gene
Pathways in cancer
Phagosome
TNF signaling pathway
Phosphatidylinositol signaling system
Inositol phosphate metabolism
Type I diabetes mellitus
Synaptic vesicle cycle
Glycerolipid metabolism
Ferroptosis
Hippo signaling pathway − multiple species
0.04
0.08
0.12
0.16
GeneRatio (SelectionCounts/SelectionSize)
SelectionCounts
2
4
6
8
10
Pvalue
0.025
0.020
0.015
0.010
0.005
